# Altered Heart Rate Variability During Gameplay in Internet Gaming Disorder: The Impact of Situations During the Game

**DOI:** 10.3389/fpsyt.2018.00429

**Published:** 2018-09-11

**Authors:** Sung Jun Hong, Deokjong Lee, Jinsick Park, Kee Namkoong, Jongshill Lee, Dong Pyo Jang, Jung Eun Lee, Young-Chul Jung, In Young Kim

**Affiliations:** ^1^Biomedical Engineering, Hanyang University, Seoul, South Korea; ^2^Psychiatry, National Health Insurance Service Ilsan Hospital, Goyang, South Korea; ^3^Institute of Behavioral Science in Medicine, Yonsei University College of Medicine, Seoul, South Korea; ^4^Psychiatry, Yonsei University College of Medicine, Seoul, South Korea; ^5^Psychiatry, Eunpyeong Hospital, Seoul, South Korea

**Keywords:** autonomic nervous system, heart rate variability, internet gaming disorder, gameplay, addiction

## Abstract

Internet gaming disorder (IGD) is characterized by a loss of control over gaming and a decline in psychosocial functioning derived from excessive gameplay. We hypothesized that individuals with IGD would show different autonomic nervous system (ANS) responses to the games than those without IGD. In this study, heart rate variability (HRV) was assessed in 21 young males with IGD and 27 healthy controls while playing their favorite Internet game. The subjects could examine the game logs to identify the most and least concentrated periods of the game. The changes in HRV during specific 5-min periods of the game (first, last, and high- and low-attention) were compared between groups via a repeated measures analysis of variance. Significant predictors of HRV patterns during gameplay were determined from stepwise multiple linear regression analyses. Subjects with IGD showed a significant difference from controls in the patterns of vagally mediated HRV, such that they showed significant reductions in high-frequency HRV, particularly during the periods of high attention and the last 5 min, compared with baseline values. A regression analysis showed that the IGD symptom scale score was a significant predictor of this reduction. These results suggest that an altered HRV response to specific gaming situations is related to addictive patterns of gaming and may reflect the diminished executive control of individuals with IGD while playing Internet games.

## Introduction

Internet gaming disorder (IGD), one of the most studied forms of Internet addiction, is characterized by a difficulty in controlling excessive Internet game use despite negative psychosocial consequences (1). Although it has not yet been fully clarified, much effort has been devoted to elucidating the neurobiological background underlying IGD (2). Some researchers are interested in the physiological features of IGD, particularly in autonomic nervous system (ANS) dysfunction. ANS dysfunction has been associated with psychiatric disorders (3), including substance abuse and behavioral addiction (4, 5). As the ANS responds to internal and external stimuli to maintain homeostasis, its function is closely related to adaptive adjustments in behavior strategies (6). ANS dysfunction likely contributes to the development and maintenance of loss of control over gaming, as individuals with IGD are unable to adjust their behavior strategies despite negative outcomes.

ANS function can be assessed non-invasively by measuring heart rate variability (HRV). A study by Lin et al. found that school-aged children with Internet addictions had lower levels of total-power HRV than non-addicted children (7). The authors also indicated that children with Internet addiction had lower high-frequency (HF) percentages and higher low-frequency (LF) percentages than non-addicted children. Data from Kim et al. similarly showed that adolescents with IGD had lower total-power HRV, but both HF and LF values were significantly lower and their ratios did not differ between those with IGD and controls (8). However, there is not sufficient evidence, with mixed results reported in previous studies, to clarify the role of ANS function in IGD.

To more accurately assess ANS function, it may be useful to measure HRV responses to particular stimuli as well as during the resting state, as this better reflects the ability of the ANS to respond appropriately and adaptively to environmental change (9). A previous study on Internet addiction showed that individuals with problematic Internet use had a lower standard deviation of the R-R interval (SDNN, reflects overall HRV levels) than non-problematic users during rest but not during and after the Trier social stress test (TSST) (10). This measure could be applied to assess HRV responses to gaming-related stimuli rather than general stress stimuli. Our previous work suggested that individuals with IGD have HRV suppression during gameplay (11). As HRV suppression may represent inefficient executive control (12), we speculated that HRV suppression in response to gaming in individuals with IGD reflects an imbalance between enhanced reward-seeking and diminished executive control (13). However, our previous study only analyzed HRV data for the first 5 min of the game. These data do not encompass the full range of responses to the variety of situations that gaming comprises, such as those requiring high levels of attention with many points to consider and those with repetitive actions or that have little influence on the final outcome of the game, which require less attention. Our previous study also did not include the subjects' perceptions of these various situations in the game. Thus, further data are needed assessing the alterations of HRV throughout the duration of the game to determine the role of the ANS in IGD pathophysiology.

Numerous studies on addiction suggest that addictive behaviors begin from a voluntary and goal-directed pattern that gradually becomes habitual and compulsive (14, 15). This progression accompanies several neurobiological changes, including weakened prefrontal cortical control and strengthened dorsal striatal control (16, 17). With this in mind, addictive gaming may also be associated with habitual and compulsive behavioral patterns. Individuals with IGD may thus be less influenced by prefrontal control, even when playing games at a high level of attention, and consequently play games in a habitual or compulsive manner rather than a goal-directed manner. To appropriately interpret these behavioral patterns, the situational factors of game should be considered.

We hypothesized that the difference in gaming behaviors between individuals with and without IGD become apparent during situations demanding a high level of attention, reflecting an addictive pattern of habitual gameplay with weakened prefrontal cortical control. We also hypothesized that HRV response during periods of high attention would be significantly related to the severity of IGD rather than to other psychological problems (e.g., depression and anxiety). Unlike the previous studies of HRV in IGD, the current study analyzed the changes in HRV during the game in which gaming addicts were actually addicted, and included consideration of the situational factors of game. Thus, in this study, we analyzed HRV of young males with and without IGD while playing an Internet game at specific time periods, including the initial and final periods, as well as during periods when the gamer was and was not particularly focused. We examined how HRV values change during these different situations, whether they differ between individuals with and without IGD, and if they are associated with the severity of IGD.

## Materials and methods

### Participants

The Institutional Review Board approved the protocol for this study (HYI-16-044), and all subjects provided signed informed consent before participating. This study included 48 subjects, all right handed and aged between 16 and 27 years (mean age: 22.0 ± 2.8 years), who were recruited through online bulletin boards, flyers, and word of mouth. All subjects were assessed for their Internet-use patterns. All subjects in this study frequently played “League of Legends” (Riot Games, 2009), the most popular multiplayer online battle arena game in Korea. To control for the difference of hedonic sensation or familiarity of each subject for the game, only those who were in the same rank in the game were recruited. Participants who primarily used the Internet for gaming and scored above 50 on the Young's Internet-addiction test (Y-IAT) (18) were classified as subjects with IGD (*n* = 21; age, 22.3 ± 2.9 years). They were reassessed for IGD via a psychiatric interview based on the criteria in the Diagnostic and Statistical Manual of Mental Disorders, fifth edition (1). Subjects who scored below 50 on the Y-IAT were classified as controls (*n* = 27; age, 21.8 ± 2.8 years).

All subjects were screened via a four-item brief screening tool (19) (feeling nervous, anxious, or on edge; not being able to stop or control worrying; feeling down, depressed, or hopeless; little interest or pleasure in doing things) to ensure they had no clinically significant depression or anxiety symptoms during more than half of the last 2 weeks. The Korean version of the Diagnostic and Statistical Manual of Mental Disorders, fourth edition, was also used to assess the presence of major mental disorders other than IGD (20) and the Wechsler adult intelligence scale IV was used to assess intelligence quotients (21). All subjects completed the following respective self-report questionnaires regarding depression, anxiety, alcohol-related problems, childhood symptoms of attention-deficit/hyperactivity disorder, and impulsivity: the Beck depression inventory (22), the Beck anxiety inventory (23), the alcohol-use disorders identification test (24), the Wender Utah rating scale (25), and the Barratt impulsiveness scale, version 11 (26).

Subjects with a substantial psychiatric comorbid condition other than IGD (e.g., depression, psychotic disorder, or substance dependence), neurological or medical disorder that affected the HRV (e.g., cardiac disease or endocrine disease), low intelligence, or were taking drugs that affected the HRV (e.g., beta blockers or anticholinergics) were excluded from the study. All subjects were psychiatric medication naïve at the time of assessment.

### Experimental protocol

**Figure 1:**
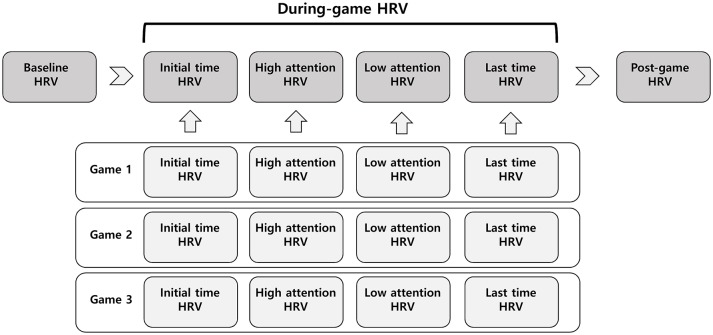
Experimental protocol of measurements for heart rate variability (HRV). Each period was 5 min.

The experimental protocol for this study is presented in Figure 1. After a rest period of at least 10 min, resting-state electrocardiograms (ECGs) were recorded for 5 min while subjects were in a relaxed sitting position for the baseline HRV. The subjects then played the online game League of Legends three times, with a 5-min rest after each game. Each game lasted for at least 20 min. After each game ended, subjects checked the game log to identify 5-min periods of high attention and low attention. For each game, initial HRV (during the first 5 min), high- and low-attention HRV (during the 5-min periods of high and low attention, respectively), and last-time HRV (during the final 5 min of the game) signals were analyzed. HRV values for each period were averaged from 3 games. After all the games were over, HRV signals were measured in the resting state for 5 min for the post-game HRV.

### HRV analysis

Three ECG channels were connected to each of the subjects' chests to obtain ECG signals via an MP150 (BIOPAC Systems Inc., Santa Barbara, CA, USA). To eliminate noise from the subjects' movement, breathing, and muscle electrical activity, the data were preprocessed using third-order Butterworth high-pass filtering with a 0.1-Hz cutoff frequency, a sixth-order Butterworth notch filter, and third-order Butterworth low-pass filtering with a 15-Hz cutoff frequency (27, 28). All ECG signals were acquired at 200 Hz, and the Pan and Tomkins method was used to automatically detect R-R intervals (29).

HRV parameters were extracted from time and frequency-domains, using a HRV analysis software (30). The time-domain method measures the time between R-R intervals or the instantaneous heart rate at a specific time. The time-domain parameters used in this study were the SDNN and the root mean squared differences of successive N-N intervals (RMSSD). SDNN indicates overall HRV, whereas RMSSD indicates short-term changes in heart rate and is used to predict parasympathetic activity (31). For the frequency-domain analyses, we used a 20% filter to remove ectopic beats, and data were interpolated at 4 Hz (32). The frequency-domain parameters were transformed with an autoregressive model. The LF domain of HRV (0.04–0.15 Hz) reflects parasympathetic and sympathetic nerve activity, whereas the HF domain (0.15–0.4 Hz) mainly represents parasympathetic activity (33). The frequency-domain HRV parameters with skewed distributions were logarithmically transformed (34). The LF/HF ratio was used as an index of sympathovagal balance (35).

### Statistical analysis

Statistical analyses were performed with Statistical Package for the Social Sciences (SPSS), version 24.0 K (SPSS Inc., Chicago, IL, USA). A *p*-value of < 0.05 was considered statistically significant. Independent *t*-tests were used to compare demographic and psychometric characteristics or absolute values of HRV parameters between subjects with IGD and controls. Repeated measures analyses of variance (ANOVAs) were used to group differences in longitudinal changes of HRV, using the period (baseline, initial, high-attention, low-attention, last-time, and post-game) as the within-subjects factor and group (IGD subjects or controls) as the between-subjects factor. Among the various parameters of HRV (lnLF, lnHF, SDNN, RMSSD, and LF/HF), we tested which parameter was statistically significant in the interaction between group and period, and *post-hoc* comparisons were applied to test whether the differences between certain periods were statistically significant. Bonferroni's corrections (*p* < 0.05/15) were conducted to adjust for multiple comparisons (15 paired comparisons of six periods). These analyses were used to characterize the patterns of HRV during gameplay (changes of specific HRV parameters between specific periods in the game) in subjects with IGD. Stepwise multiple linear regression analyses were then conducted to identify significant predictors of these patterns. For these analyses, demographic or psychometric characteristics (Y-IAT, age, intelligence quotient, scores from self-report questionnaires) of all subjects were entered as independent variables.

## Results

### Clinical characteristics of the subjects

Subjects with IGD had Y-IAT scores that were significantly higher than the controls (*p* < 0.001; Table 1). Subjects with IGD and controls did not differ significantly in self-reported scale scores for depression, anxiety, alcohol-related problems, and childhood attention-deficit/hyperactivity symptoms. However, subjects with IGD scored significantly higher on tests of impulsivity than did controls (*p* = 0.003).

**Table 1:** Demographics and clinical variables of subjects.

**Variable**	**Control**	**IGD**	***p*-value**
	**(*n* = 27)**	**(*n* = 21)**	
**DEMOGRAPHICS**			
Age, years	21.8 (2.8)	22.3 (2.9)	0.494
Education level, years	12.1 (0.4)	11.8 (0.8)	0.103
Full-scale intelligence quotient	110.1 (10.5)	110.7 (12.0)	0.953
**PSYCHOLOGICAL FACTORS**			
Young Internet-addiction test	30.8 (11.6)	63.1 (8.8)	<**0.001**
Beck depression inventory	7.0 (4.9)	9.5 (7.9)	0.285
Beck anxiety inventory	5.6 (4.5)	5.7 (4.4)	0.944
Barratt impulsiveness scale	49.9 (5.5)	56.4 (7.8)	**0.003**
Alcohol-use disorder identification test	10.6 (5.6)	10.8 (7.4)	0.953
Wender Utah rating scale	28.5 (12.5)	24.3 (14.6)	0.232

*Data are presented as means (standard deviations)*.

*IGD, Internet gaming disorder*.

*Bold value means that p-value is less than 0.05*.

### HRV changes

For all periods, there were no statistically significant differences between subjects with IGD and controls in any of the HRV parameters (Table 2). However, interaction effects between period and group were statistically significant for lnHF (*p* = 0.043) and RMSSD (*p* = 0.028) via repeated measures ANOVAs (Table 3).

**Table 2:** Absolute values of HRV parameters in subjects.

**Parameter**	**Baseline HRV**			**Initial-time HRV**			**High-attention HRV**			**Low-attention HRV**			**Last-time HRV**			**Post-game HRV**		
	**Control**	**IGD**	***p*-value**	**Control**	**IGD**	***p*-value**	**Control**	**IGD**	***p*-value**	**Control**	**IGD**	***p*-value**	**Control**	**IGD**	***p*-value**	**Control**	**IGD**	***p*-value**
LnHF	4.3 (0.9)	4.7 (0.7)	0.079	4.4 (0.7)	4.3 (0.7)	0.764	4.3 (0.8)	4.2 (0.7)	0.535	4.4 (0.8)	4.3 (0.6)	0.899	4.3 (0.8)	4.3 (0.6)	0.977	4.2 (0.8)	4.5 (0.7)	0.182
LnLF	5.7 (0.8)	6.0 (0.6)	0.164	5.8 (0.7)	5.6 (0.5)	0.269	5.8 (0.6)	5.6 (0.6)	0.267	5.8 (0.7)	5.6 (0.5)	0.345	5.7 (0.7)	5.7 (0.5)	0.697	5.7 (0.9)	5.8 (0.5)	0.564
SDNN	45.3 (22.8)	50.3 (14.5)	0.386	48.4 (16.0)	46.5 (14.2)	0.677	47.7 (16.6)	44.6 (13.3)	0.493	49.3 (17.4)	45.9 (13.3)	0.463	48.2 (19.0)	45.3 (14.7)	0.571	45.0 (20.0)	50.1 (13.9)	0.326
RMSSD	35.3 (22.2)	40.7 (19.5)	0.387	36.3 (16.1)	32.5 (11.4)	0.371	35.7 (16.7)	31.2 (12.7)	0.311	35.7 (17.3)	32.3 (11.6)	0.448	35.3 (18.4)	31.4 (13.1)	0.424	32.3 (18.3)	38.1 (17.0)	0.262
LF/HF	4.6 (2.7)	4.6 (3.7)	0.967	4.6 (1.9)	4.0 (1.6)	0.253	4.9 (2.5)	4.7 (2.6)	0.859	4.8 (2.3)	4.2 (2.0)	0.336	4.7 (2.0)	4.5 (1.8)	0.681	5.5 (3.5)	4.9 (4.8)	0.656

*Data are presented as means (standard deviations). HRV, heart rate variability; lnLF, natural logarithm of low frequency; lnHF, natural logarithm of high frequency; RMSSD, square root of the mean of the sum of the squares of differences between consecutive normal-to-normal intervals; SDNN, standard deviation of the normal-to-normal interval*.

**Table 3:** Repeated measures ANOVA for HRV parameters.

**Parameter**	**Source**	**${}_{\text{p}}^{\text{2}}$**	***F***	***p*-value**
LnHF	Group	0.004	0.163	0.689
	Period	0.303	3.651	**0.008**
	Period × Group	0.232	2.541	**0.043**
LnLF	Group	0.001	0.046	0.831
	Period	0.113	1.074	0.389
	Period × Group	0.192	1.999	0.099
SDNN	Group	< 0.001	0.002	0.967
	Period	0.109	1.031	0.412
	Period × Group	0.183	1.884	0.118
RMSSD	Group	0.001	0.025	0.875
	Period	0.215	2.306	0.061
	Period × Group	0.251	2.814	**0.028**
LF/HF	Group	0.008	0.392	0.534
	Period	0.114	1.078	0.387
	Period × Group	0.083	0.759	0.584

*${}_{p}^{2}$, effect size as partial eta-squared. HRV, heart rate variability; lnLF, natural logarithm of low frequency; lnHF, natural logarithm of high frequency; RMSSD, square root of the mean of the sum of the squares of differences between consecutive normal-to-normal intervals; SDNN, standard deviation of the normal-to-normal interval*.

*Bold value means that p-value is less than 0.05*.

*Post-hoc* comparisons showed that subjects with IGD had significant reductions in lnHF for high-attention HRV (*p* = 0.001) and last-time HRV (*p* = 0.003) compared with that for baseline HRV. Although not statistically significant after correction for multiple comparisons, subjects with IGD also had trends toward reductions in RMSSD for high-attention HRV (*p* = 0.007) compared with that for baseline HRV. Unlike subjects with IGD, subjects without IGD did not show significant differences in any of the HRV parameter between specific periods (*p* > 0.05).

Stepwise multiple linear regression analyses were performed with the differences of lnHF for high-attention HRV and last-time HRV relative to baseline HRV as the dependent variables (Table 4). Of the independent variables, only the Y-IAT score was a significant predictor of the difference of lnHF between high-attention HRV and baseline HRV. However, the Y-IAT score and Beck depression inventory score were significant predictors of the difference in lnHF between last-time HRV and baseline HRV. All the variables used for multiple linear regression had a variance inflation factor of < 5.

**Table 4:** Stepwise multiple linear regression analysis with HRV features.

**Independent variables**	**β**	***t***	***p*-value**
**With difference in lnHF between high-attention and baseline HRVs as dependent variables**			
Age	−0.510	**-**0.368	0.714
Full-scale intelligence quotient	−0.226	−1.684	0.099
Young Internet-addiction test	−0.378	−2.766	**0.008**
Beck depression inventory	0.256	1.848	0.071
Beck anxiety inventory	0.032	0.234	0.816
Barratt impulsiveness scale	0.059	0.374	0.710
Alcohol-use disorder identification test	0.110	0.803	0.426
Wender Utah rating scale	−0.090	−0.653	0.517
*R*^2^ = 0.143; adjusted *R*^2^ = 0.124; SEE = 13.6
**With difference in lnHF between last-time and baseline HRVs as dependent variables**			
Age	−0.145	−1.046	0.301
Full-scale intelligence quotient	−0.179	−1.306	0.198
Young Internet-addiction test	−0.369	−2.611	**0.012**
Beck depression inventory	0.308	2.178	**0.035**
Beck anxiety inventory	−0.087	−0.533	0.596
Barratt impulsiveness scale	0.030	0.187	0.853
Alcohol-use disorder identification test	0.069	0.490	0.627
Wender Utah rating scale	0.082	0.584	0.562
*R*^2^ = 0.169; adjusted *R*^2^ = 0.132; SEE = 0.5

β, standardized regression coefficient. HRV, heart rate variability; lnHF, natural logarithm of high frequency.

*Bold value means that p-value is less than 0.05*.

## Discussion

In this study, we measured the changes in HRV in young males with IGD while they played an Internet game. Although the absolute values for HRVs did not differ between young males with IGD and controls, those with IGD had significant differences with respect to longitudinal changes of lnHF and RMSSD during gaming. Specifically, lnHF was reduced from baseline during periods of high attention and during the last 5 min of the game, supporting our hypothesis that a characteristic HRV response would become apparent during game periods requiring a high level of attention. Further analyses supported our second hypothesis, showing that the severity of the IGD, assessed by the Y-IAT score, predicted this characteristic reduction in lnHF.

The group differences in patterns of change in lnHF and RMSSD we observed during gameplay are consistent with a previous study, suggesting that HRV responses in subjects with IGD differ from those of controls while playing the game (11). HF and RMSSD are closely correlated and reflect vagal activity (35). The neurovisceral model suggests that vagally mediated HRV is an index that reflects executive control over affective and cognitive processes (36). According to this model, these differences are related to the difficulty in exerting executive control over excessive gaming in subjects with IGD.

Subjects with IGD showed significant reductions in lnHF for high-attention HRV and last-time HRV compared with that for baseline HRV. Although statistical significance was not high enough, they also showed trends toward reductions in RMSSD for high-attention HRV compared with that for baseline HRV. A large reduction of vagally mediated HRV is suggested to be a specialized HRV feature that reliably reflects weakened prefrontal neural function responsible for top-down executive control (37). In the present study, the subjects determined what period required high attention, and the gameplay during the final 5 min was a determinant of whether the game was won or lost. Thus, during these important periods, the reduction in HF-HRV suggests that top-down executive control was diminished when the demand for concentration on the game was increased. We infer that these characteristics of gameplay in young males with IGD are related to their habitual/compulsive game use.

Interestingly, the only predictor of the reduced lnHF during periods of high attention was the severity of the IGD, suggesting that an altered HRV response to gaming in individuals with IGD reflects the addictive features of gaming. Therefore, the current findings support a putative role of altered ANS response to gaming in the status of IGD. On the other hand, the severity of both IGD and depression predicted the reduction in lnHF during last 5 min of the game, when some of the gamers may have given up. We speculate that psychological characteristics of depression affect the extent to which subjects concentrate at the end of the game, which is associated with their HRV responses.

As psychological conditions (e.g., depression and anxiety) were assessed prior to participation, we ensured that the subjects with IGD and controls did not differ in this regard. This may explain why no significant differences in the resting-state HRVs were observed. However, subjects with IGD self-reported higher impulsiveness than controls, in agreement with previous studies in which high impulsivity is the prominent psychobehavioral feature in IGD (38, 39). Moreover, high impulsivity is related to diminished executive function (40). Nevertheless, impulsivity and executive function each contain various components, and the relationship between them is complex (41). Further research is needed to clarify the association between the high impulsivity of individuals with IGD and their HRV responses to gaming.

There are some limitations to this study. First, the sample size of this study is small, thus not all variables affecting HRV could be controlled. Second, the inclusion of indexes besides HRV is needed to fully reflect the subjects' physiological and autonomic responses to gaming. Third, the design of this study was cross-sectional; therefore, the changes in HRV responses observed during gameplay should be followed up in longitudinal studies of subjects with IGD. Such studies would validate the use of HRV as an important index reflecting the progress of treatments for IGD.

Despite its limitations, our current study is the first to analyze HRV data during the game, encompassing responses to the specific gaming situations. Young males with IGD showed vagally mediated HRV responses to gaming that were significantly different from those of healthy controls, particularly during periods requiring high attention. They showed a reduction in HF-HRV that was also related to the severity of their IGD. This alteration in their autonomic responses suggests that individuals with IGD are less influenced by executive control, even when playing games at a high level of attention. Our results suggest that the habitual gaming behaviors of individuals with loss of control over gaming reflects diminished prefrontal control.

## Ethics statement

All of the procedures involving human participants were performed in accordance with the ethical standards of the institutional and national research committees and with the 1964 Helsinki declaration and its later amendments. The experimental protocol was approved by the Institutional Review Board at Hanyang University (HYI-16-044).

## Author contributions

Y-CJ and IK contributed to the study design. JP and JEL collected the clinical and bio-signal data. SH and DL performed statistical analysis and wrote the first draft of the manuscript. KN, JL and DJ provided critical revision of the manuscript and important intellectual content. All authors contributed to manuscript revision, read, and approved the submitted version.

### Conflict of interest statement

The authors declare that the research was conducted in the absence of any commercial or financial relationships that could be construed as a potential conflict of interest.
